# Decision-making by medical officer in charge during major incidents: a qualitative study

**DOI:** 10.1186/s13049-021-00937-8

**Published:** 2021-08-21

**Authors:** Karin Hugelius, Monica Rådestad, H. Al-Dhahir, L. Kurland

**Affiliations:** 1 Faculty of Medicine and Health, Örebro University, 70182 Örebro, Sweden; 2 Department of Clinical Science and Education, Karolinska Institutet, SödersjukhusetStockholm, Sweden; 3 Capio St. Görans Hospital, Stockholm, Sweden; 4 Department of Emergency Medicine, Örebro University Hospital, Örebro, Sweden; 5 Department of Medical Sciences, Örebro University, Örebro, Sweden

**Keywords:** Decision-making, Incident command major, Incidents, Major incident management

## Abstract

**Background:**

An incident command structure is commonly used to manage responses to major incidents. In the hospital incident command structure, the medical officer in charge (MOC) is in a key position. The decision-making process is essential to effective management, but little is known about which factors influence the process. Therefore, the current study aimed to describe factors influencing decision-making of MOCs.

**Methods:**

A conventional content analysis was conducted based on 16 individual interviews with medical doctors who had been deployed as MOCs at Swedish hospitals during major incidents.

**Results:**

The results showed that the decision-making and re-evaluation process was a comprehensive analysis influenced by three categories of factors: event factors, including consequences from the type of event, levels of uncertainty and the circumstances; organizational factors, including the doctor’s role, information management and the response to the event; and personal factors, such as competence, personality and mental preparedness.

**Conclusions:**

Reliable and timely information management structure enabling the gathering and analysis of essential information, a clear command structure and appropriate personal qualities were essential and contributed to successful MOCs decision making in major incidents.

## Background

Crisis management is a systematic approach to averting crises and effectively managing those that do occur [1]. Major incidents (Mis) include different events, from large accidents, terrorist attacks and earthquakes to failure of hospital infrastructure (e.g. electric power and emergency power, water, medical gases and technological systems). An MI can therefore be defined as an ´event that is so extensive and demanding that resources have to be organized, managed and used in a special way´ [2]. Crisis management needs therefore to apply an “all hazards” approach [3].

The healthcare system must be adaptive during the response to an MI by adjusting its organization, methods and ethical principles. The medical system—including prehospital emergency services, hospitals and primary healthcare providers—must increase its capacity on short notice and switch into a response mode, which requires a well-functioning command structure that is capable of adapting patient flows, medical care and other attendant adjustments. At the same time, the system must be prepared to manage a situation in which the hospitals themselves may be threatened or damaged by the incident [4]. Managing a complex and dynamic situation, such as a major incident, requires adjusting organizational and medical procedures at both individual and organizational levels [5]. The design of such response strategies varies, a command structure, which differs from the everyday organizational structure, is typically implemented in accordance with the disaster management plan when a major incident alert is activated [6].

The incident command system is commonly used worldwide by fire departments, police organizations, and military agencies, as well as in hospital responses to major incidents [7]. This system employs a logical, scalable management structure that incorporates defined responsibilities, clear information-sharing channels and a distinct nomenclature. It relies on organizational positions and roles, rather than on individuals, and is designed to be usable regardless of the type of major incident or the time of day [6, 7]. A key component in the Swedish health care incident command system on the local- or regional level is the medical officer in charge (MOC) available 24/7 [6, p. 83]. The MOC leads the first phase of the hospital response. Many hospitals use one of the senior physicians on call, typically from internal medicine, surgery or anesthesia, alongside with an incident commander. The MOC is responsible for making comprehensive medical decisions and prioritizations, which lead the overall medical response to the incident. There is no formal, general description on competences required to act as MOC in Sweden. Therefore, the formal competence, skills and training may vary. In the current study, a MOC was defined as the function within the hospital command structure that is entitled to make strategic medical decisions, concerning care in either the emergency department or in the hospital or the regional incident command group, depending on the organization.

Decision-making is key to an effective crisis response [4]. In crises, decision-making is time-sensitive and based on uncertain information and vague situational awareness [8]. Additionally, decision-making in crises is influenced by previous knowledge and experience; the extent to which the specific situation is recognized (e.g., in its severity and hazardousness); the quality, amount and speed of the data available; and the responders’ ability to integrate information into their mental frameworks [9]. The predominant theory in this field is naturalistic decision- making [10]. This approach focuses on decision-making as a cognitive process, including sense-making, situational awareness and planning- under difficult conditions [11]. In contrast to such logical, cognitive models for crisis decision-making, emotions have been suggested not only to contribute to making successful decisions but also to serve as an essential element in intuitive decision-making processes [12].

Although the decision-making process has been the focus of prior research interest [8], less attention has been paid to which components that influence critical decisions in a medical response organization during a major incident. Such knowledge is essential to understanding both the processes and outcomes of decision-making and being able to optimally organize and structure the management and command structures.

## Methods

### Aim

This study aimed to describe factors influencing decision-making of MOCs.

### Design

A qualitative descriptive study design using semi- structured interview was used.

### Participants

Interviews were conducted with 16 medical doctors who had been MOCs [6] during a medical response to a major incident. The MOCs were required to have had the authorization to make comprehensive, strategic medical decisions during the primary response phase in an emergency department or as a medical commander in a hospital command group for inclusion as a participant. In addition, the participants should also have been deployed in a local or regional incident command organization within the Swedish healthcare system, following a major incident or other major event leading to a partly or complete activation of the disaster management plan. No formal training was required to participate, since the regional differences on requirements vary. The study participants were recruited by snowball sampling [8] or through contact with disaster preparedness coordinators, who mediated contact with potential study participants. The authors were guided by five items having an impact on information power: (1) study aim, (2) sample specificity, (3) use of established theory, (4) quality of dialogue, and (5) analysis strategy [13]. A total of 16 medical doctors agreed to share their experiences in the interviews. All the participants were provided with written and verbal information about the study before the interviews.

### Data collection procedures

All interviews were conducted via telephone or Skype by one of the research team members (HA). A semi-structured interview guide was used (see Table 1). This guide was developed specifically for the current study and was tested via a pilot interview. Minor changes were made after the pilot interview, which was itself considered a valid source of data and was included in the analyzed material. All interviews were recorded and transcribed verbatim.

**Table 1:** Interview guide

Can you describe an event in which you made strategic decisions?*
How did you get the information about the major incident?*
What function did you have during the major incident?*
Were there any checklist or action plans that specified your responsibilities and decisions to be made?
What were you trying to achieve with the decisions you made?
Can you give an example of a decision you made during a major incident?*
What do you think influenced or could have influenced your decision-making process?*
What information was needed to make these decisions?
What were the main challenges when managing the major incident?
Do you have specific training in acting as a MOC and in making this type of necessary strategic decisions?
Faced with a similar situation in the future, would you do anything differently?

Note that only questions marked with * were used in all interviews

### Analysis

Conventional content analysis [14] was employed to analyze the data. After reading each entire interview transcript several times, meaning units relevant to the research question were extracted and subsequently sorted into themes, subcategories and categories. No separate condensation was made when the meaning units appeared relatively short and clear. Separate notes on the researcher’s (KH) immediate impressions from the analysis were also made and contributed to the final results [14]. The analysis was performed by KH and MR. LK and HA verified the analysis by reading through all the original units in relation to the results.

## Results

Of the 16 MOCs, 11 were men and five women between 36 and 68 years old (mean 58 yeas). All were medical doctors with different specialties covering anesthesia (n = 7), (surgery (n = 4), internal medicine (n = 4), and radiologist (n = 1). The interviews lasted from 10 to 35 min (mean; 24 min). The incidents the MOCs had experienced were bus accidents, terrorist attacks, traffic accidents, hospital fires, power shortages and interruptions in hospital medical record systems. The major incidents had occurred between one and 10 years ago. Many of the interviews covered more than one event.

The main theme, decision-making and re-evaluation process consisted of three categories (event factors, organizational factors and personal factors) further divided into nine subcategories, which influenced the decision-making and re-evaluation process. Table 2 shows an overview of these findings.

**Table 2:** Overview of themes, categories and subcategories

Main themes	Decision-making and re-evaluation process		
Categories	Event factors	Organizational factors	Personal factors
Subcategories	Type of event	Role	Competence
	Level of uncertainty	Information management	Personal qualities
	Circumstances	Response	Mental preparedness

### Event factors

Several factors relating to the major incidents themselves influenced the MOCs’ decision-making. Information on the type of event—such as whether it was a bus crash, a terrorist attack or water contamination within a hospital—was used to estimate the types of injures to be expected. Circumstances—such as the time of day, the time of year, the weather and the location of the event—were used to analyze the expected consequences: the number of affected and injured people and possible medical consequences, such as the risk of hypothermia. Some circumstances were believed to mitigate the consequences of a major incident. An example is when the event occurs in the daytime, especially between shifts when the healthcare system has a better chance of coping with the potential influx of a large number of victims. The reverse was true if the event took place at an unsuitable time, such as during the night or on a weekend. Hence, it was not the events themselves but their expected consequences that influenced the decision-making process.

It was in the middle of the night. Then they called at three ... three AM or four AM at night. Between Saturday and Sunday. And it was not a good time. It was a bad time. It was pretty hard. It was difficult to get people to come, of course. (Participant 1)

The level of uncertainty varied during the decision- making- and management process of specific events. It was most often high in the early state of most events due to limited or unconfirmed information. Sometimes, the first notification came through the media or via personal reports from ambulance personnel on site. Difficulties confirming the number of victims in the early stages after the event caused feelings of uncertainty and frustration.

The biggest challenge in this whole event was for me an uncertainty about the inflow of patients to the hospital. There were uncertainty and ambiguity about the number of victims…. (Participant 5)

In other situations, the uncertainty was related to what had actually happened or where the event had taken place. Rumors of several events at the same time caused problems with relying on the information received and estimating the consequences. To interpret the information and estimate the consequences, the MOC used a form of triangulation to confirm the received information by including information from formal reports, media reports and personal contacts and colleagues, both in the early stages after the event or as the event unfolded.

### Organizational factors

The role or function of the MOC in a disaster management organization influenced the quality of decision-making and how detailed the decisions were. In the very early stages of the incident response, decisions tended to be more operational or tactical; later, they became more strategic. Some MOCs expressed that they made both operational and strategic decisions at the same time. Awareness of the MOC’s role in the hierarchy and command system and being clear on the mandate of the MOC were considered important to making rapid, accurate decisions. Keeping a distance from the clinical, operational level was difficult at times but necessary for maintaining an overview of the situation and enabling the conducting of analysis before making decisions.

When I have made such decisions, I have typically not been in the designated function according to the disaster management plan … because that person has not been in place or did not have the right skills for doing it. After those experiences, we have changed the plan and we hope this one fits better with how you should … actually work…. (Participant 4)

Information management was crucial to the decision-making process. All interviewees expressed the need for accurate, timely information. The challenges included difficulties acquiring confirmed information from external stakeholders, such as the fire brigade or technical experts, or from inside the medical organizations, such as from different departments or units.

You must have very good information. This is more about when you get the information, which can sometimes take longer than you would like. But to get all the information and then you have to focus on the information you have, and then maybe it was not the whole picture but then you have to make a decision and then when you get the full data, you have to revise it. (Participant 14)

The internal information flow, within the medical response and command organization, was important not only for obtaining information but also for spreading that information from the MOC to other medical professionals in the response organization. Difficulties disseminating decisions and strategies led to a lack of trust between the MOC and other health care personnel as well as to problems, causing the MOC to follow-up and re-evaluate the decisions.

From this staff meeting when decisions were made under the overall commander, it was not clear how information about the situation and about the overall decisions, would be disseminated in the organization. So to all different clinics that are active during on-call time, I did not know how it would go and…. It was probably not clear how it would happen either. (Participant 13)

The response from the healthcare system also influenced the decision-making process in many ways. Sometimes, the response reduced the negative consequences of the event to such an extent that further decisions were not needed. It was, therefore, necessary to analyze the effects of the response from both short- and long-term perspectives to make wise, timely decisions. A lack of information from the internal organization, such as not getting a clear picture of what was going on in the emergency department, blurred the situation and made it difficult to value the response actions. In some instances, the influx of medical professionals willing to be deployed in the response organization or to take part in the command organization necessitated the reduction of such initiatives when they were considered not to be helpful but to place extra stress and burden on the response organization.

### Personal factors

The MOCs were required to have specific competence that included knowledge about not only the medical consequences of specific events but also disaster medicine principles in general, crisis management and the structure of the response organization. Skills working in a command system were considered essential, but skills in communication and the analysis of complex situations were also necessary. Personal experience from previous incidents was used to analyze the consequences and foresee various scenarios for the development of the situation. Such personal experience also led the MOCs to be confident in their decision-making, which reduced stress and made them more comfortable in their roles.

There are people who talk about black swans. That is, unusual events. And very many of the events that we have handled can be considered very unusual.... We have to deal with them anyway… and therefore it is important to have a general competence. It is more important than a checklist. (Participant 6)

MOCs’ personal qualities also influenced decision-making. Preferably, decision-makers should have a high tolerance for stress and dealing with uncertainty. They should also have the ability to stay calm and focused in difficult situations. In these cases, the MOCs were required to be open-minded, not only to take in the manifest information as reports but also to listen to and understand the latent information given—for example, the level of stress of the person giving the report, based on the sound of his or her voice. Therefore, the ability to use an appropriate level of sensitivity and maintain that sensitivity in stressful situations was considered an important personal attribute. Considerable courage was also needed to make decisions based on vague information and with reduced clarity as to the consequences. The participants emphasized that making decisions during a major incident or disaster is very different from everyday work. Even if the MOC was accustomed to working in a high-stress-level environment, such as the intensive care or the emergency department, acting as a MOC was different. The MOC could not only rely on contingency plans and checklists but needed to be able to improvise. The decision-maker also needed to be prepared to feel uncomfortable while still making the necessary decisions.

Then when you make a decision, you have to stand for it. And then you have made a decision and then you notice that it was not right, then you have to have so many options left….But you have to have the courage to do things. (Participant 2)

Being mentally prepared was a key to successful decision-making. If unprepared, the MOC would not be able to manage his or her own stress reactions or the complexity of the situation. There was also a risk that the MOC would not make any decisions at all, which was mentioned as being the worst possible outcome.

I don’t really think you can prepare for something like this. As I said, you probably do not know how to react. It is certainly a matter of personality in many ways and you do not know it either. It is more hypothetical for those who have not been involved in such a disaster situation. There are certainly those who have taken a lot of courses and are prepared and believe themselves to be, so to speak, adequately prepared who may not be able to do it and then others who are completely novice who prove that this, this went very well…. (Participant 16)

It was difficult to imagine how to function as a MOC without personal experiences from major incidents, which made the role challenging. Sometimes, the MOCs were surprised by the kinds of decisions they were required to make or by how vague the available information was, even if they had heard about these things when in training. Another aspect of the MOCs’ mental preparedness was being ready to work in the emergency department or the command group among new colleagues with whom they had never worked before. The ability to work as a team was considered essential for effective disaster management.

My point of view is that it is very important to practice, and to have a good training in staff work. It is absolutely basic and it is absolutely important for the person who is the medical officer in charge. It is important that, as a medical officer in charge, you also have a pretty good idea of how to work with staff. But practice, practice, practice…. You can’t overemphasize the value of the exercise in this kind of thing.... (Participant 1)

The MOC’s role is a difficult and essential task, and everyone who holds the MOC position should be trained for this role.

### The decision-making and re-evaluation process

**Fig. 1:**
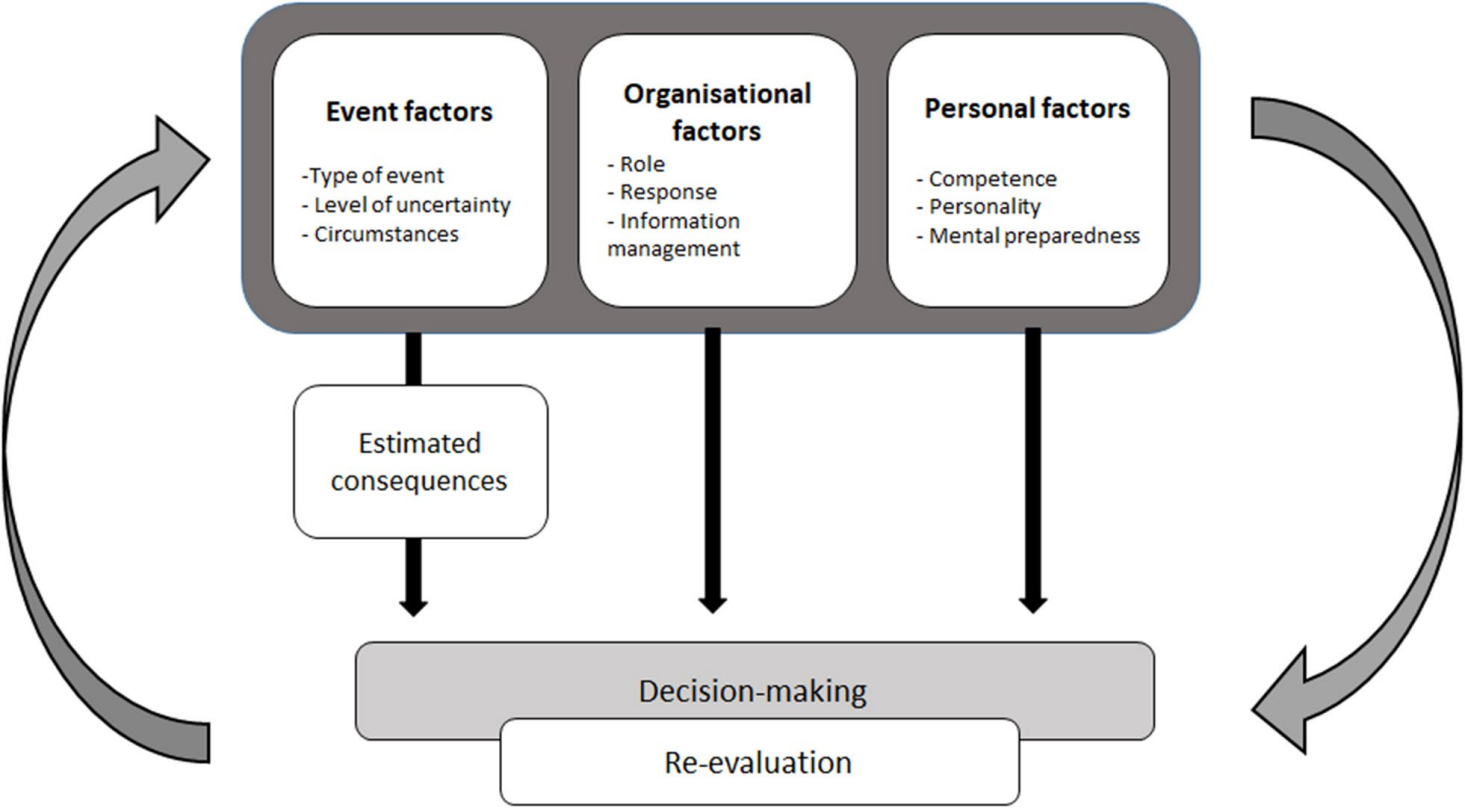
Process of decision making among medical officers in charge based on themes, categories and subcategories

The main theme, decision-making and re-evaluation process described a comprehensive construction where all the above mentioned factors, i.e. event related factors, organizational factors and personal factors, were integrated (see Fig. 1). Most often, the MOC made decisions on his or her own, but, sometimes, a short collaborative discussion with senior physicians in the command organization occurred before definitive decisions were made. Such collaborative discussions, even if very brief, were believed to improve the quality of the decisions and ensured that the decisions were aligned with other activities and the overall strategy of the disaster management organization. The re-evaluation of decisions was an ongoing process throughout the responses, and part of the decision-making process by ensuring that all decisions were dynamic and could be changed. Re-evaluation was largely based on reliable information but was also on the event as it developed as well as organizational and personal factors. The participants emphasized that decisions need to be flexible. At the same time, a change of decision was tough to disseminate across the organization and was, therefore, considered less effective.

## Discussion

The current study has shown that event-related factors, including their consequences, organizational factors and personal factors, influenced the decision-making and re-evaluation process among MOCs during major incidents. Clarity of the event and its consequences, a clear role and mandate of the MOC, structured information management process, personal competence including specific knowledge and skills of major incident management as well as previous experiences, personal qualities tolerance for stress, mental preparedness and a constant re-evaluation of the situation were necessary for decision making.

Among the event-related factors, the type of event but also the level of uncertainty and circumstances were used to estimate the consequences from the event and to adapt the response to that. Previous studies have suggested that factors influencing decisions concerning the distribution of victims in mass casualty situations include, for example, the number of injured, the types of injuries, the available resources and the skills of the responders [14]. In the current study, it was clear that such event-related information was initially unreliable and, in most events, unavailable for various periods of time. Uncertainty is a central component in many decision-making models, including the naturalistic decision-making approach [11]. Other related concept such as risk or ambiguity has also been studied from a crisis’s decision-making perspective [8]. The literature suggests two situations that case uncertainty in a naturalistic decision- making process; lack of information or different information on the same thing [8]. Both these situations were experienced by the MOC´s in this study. A gap between the expected or requested information and the available information complicated the decision-making. Information management, a separate process within the organizational factors found to influence decision-making, was key for effective decision-making. Information management in crises has been described as a cycle of processes: identifying information needs, collecting adequate information, analyzing that information by organizing it and, finally, disseminating the information to internal and external partners [16]. Too much information can overcrowd communication channels and reduce the information processing capacities of the decision-makers [17]. This oscillation is a common and well-known challenge in crises management and can be mitigated by using a structured information management methodology [16]. However, no participant in the current study spontaneously brought up the information management as a structured process within the command organization. There were also no reflections on the specific information management skills required to act as a MOC. This fact indicates the necessity of further implementation of adequate information management processes in the command structure and raising awareness of the requirements and processes behind effective information management during crises among MOCs. The knowledge and skills to maintain an effective information management process among MOCs is of interest for future studies.

Another organizational factor was the role of the MOC. When establishing a temporary incident command structure, the MOCs switched roles and responsibilities from their everyday duties. Knowledge of the command structure and the mandate for their role as MOC was important for the decision-making process. Clarity of role has been found to be an essential mechanism in crisis management, especially in organizations and teams that do not usually work together [17, 18]. Separating the strategic and operational command levels has also been found to be essential [1]. When describing their experiences, the MOCs in the current study stated that they sometimes oscillated between making strategic and operational decisions. This could indicate confusion in the organizational setting concerning the levels of command and could present a challenge for the individual in keeping to his or her designated role and level in the command structure.

The personal factors found to influence decision-making included specific knowledge, experiences and skills but also personal qualities and mental preparedness. Having adequate situational awareness is fundamental for decision-making during crises [19]. However, situational awareness implies having not only access to the actual numbers and data but also the personal competence to analyze the potential consequences and impact of such data [19]. As found in the current study, being able to work intuitively, rather than with exact information, is an important skill among MOCs [9, 20]. To be able to balance the need to stay within the command structure and, at the same time, use a certain amount of improvisation to deal with unexpected developments was also highlighted by the study participants. Previous personal experiences and the capacity to improvise have also been found to facilitate decision-making in crises [21]. Developing such complex personal competence from unusual situations takes time. Using the same disaster management system and the disaster management plan structure in every incident, even though event types and circumstances differ, enables the system and the individuals involved in it to build competence, experience and resilient responses [22]. Exercises and training can also play an important role in developing the necessary personal skills and competencies [23] and were emphasized by the study participants. It does not help to have good planning and organization if education and training for staff in specific positions (e.g., command positions, such as MOC) are neglected or insufficient. The key of major incident responses is the ability to make rapid and accurate decisions also in situations with vague or minimal information.

The question concerning the importance of having previous experience from MIs is interesting since mistakes in disaster management tend to be repeated [17, 24]. Repeating training is necessary, since MIs are uncommon, especially from the perspective of the individual´s experience. With that in mind, staff in specific command positions need to train and practice decision- making in major incident response, for example in simulations or tabletop exercises. The requirements of training of decision- making may also add value to the previous experience from major events and make it easier to build mental preparedness. Much of the decision-making in disaster response is about managing uncertainty and ambiguity. Tolerance for ambiguity in crisis decision-making has not been found to correlate with profession, professional experience or age [8]. This fact strengthens the finding that personal qualities might be of greater importance for decision-making in major incidents than the actual profession or position of the decision-maker in the everyday organization.

### Limitations

The current qualitative study relied on interviews with a limited number of Swedish physicians who had served as MOCs and had a limited amount of experience with major incidents. As MIs occur infrequently many physicians man never be exposed to major incidents. Thus, the transferability of the results could be questioned. However, the experiences expressed covered several types of events, different geographical areas and both local and regional commands. It was not possible to determine how many years each participant had been able to be called in as a MOC, due to different on call systems in different regions. Many of the interviewees also described different experiences. The length of the interviews varied. However, the impression from the interviewer and the other authors were that also the shorter interviews provided insights and rich data. The participants managed to describe their experiences on the subject within the allotted time. What they could share on the subject within the interview period was assessed to fulfill the criteria for information power [13]. The theoretical concept naturalistic decision making has been used as theoretical framework for the study. There is, therefore, no reason to believe that the decision-making process in other emergency contexts would differ significantly/ to a large extent from these experiences. The data analysis was conducted by one author (KH) and was subsequently verified by an author with a deep understanding of the full text of the interviews (MR) and the author who conducted the interviews (HA). The fourth author (LK) verified the results. All authors have rich professional experience in disaster medicine and disaster management. By processing the data this way, they could reflect on their individual preunderstanding and its potential impact on the analysis process and results [8].

## Conclusions

This study showed that factors related to both the occurred event, the crises organization and personal attributes of the MOC influenced the decision-making process among MOCs in the hospital crises response during major incidents. Reliable and timely information management structure enabling the gathering and analysis of essential information, a clear command structure and appropriate personal qualities were essential and contributed to the decision making of MOCs in major incidents. We suggest that the results be used to influence the selection of MOCs and to raise awareness of the complexity in the decision-making process in addition to supporting training in decision making, so that individuals can be trained to serve in command positions during major incidents.

## Data Availability

The data used and analysed during the current study are available from the corresponding author on reasonable request.

## Abbreviations

MI: Major incidents
MOC: Medical officer in charge
